# Tomato UV-B receptor SlUVR8 mediates plant acclimation to UV-B radiation and enhances fruit chloroplast development via regulating SlGLK2

**DOI:** 10.1038/s41598-018-24309-y

**Published:** 2018-04-17

**Authors:** Huirong Li, Yuxiang Li, Heng Deng, Xiaochun Sun, Anquan Wang, Xiaofeng Tang, Yongfeng Gao, Ning Zhang, Lihuan Wang, Shuzhang Yang, Yongsheng Liu, Songhu Wang

**Affiliations:** 1 Ministry of Education Key Laboratory for Bio-resource and Eco-environment, College of Life Science, State Key Laboratory of Hydraulics and Mountain River Engineering, Sichuan University, Chengdu, 610064 China; 2 CAS Center for Excellence in Molecular Plant Sciences, Chengdu Institute of Biology, Chinese Academy of Sciences, Chengdu, 610041 China; 3 School of Biotechnology and Food Engineering, Hefei University of Technology, Hefei, 230009 China; 4 School of Life Science and Engineering, Southwest University of Science and Technology, Mianyang, Sichuan 621010 China; 5 Shaanxi University of Chinese Medicine/Shaanxi Collaborative Innovation Center of Chinese Medicinal Resources Industrialization, Shaanxi Sheng, China

## Abstract

Plants utilize energy from sunlight to perform photosynthesis in chloroplast, an organelle that could be damaged by solar UV radiation. The ultraviolet-B (UV-B) photoreceptor UVR8 is required for UV-B perception and signal transduction. However, little is known about how *UVR8* influence chloroplast development under UV-B radiation. Here, we characterized tomato *UVR8* gene (*SlUVR8*) and our results indicated that *SlUVR8* facilitate plant acclimation to UV-B stress by orchestrating expression of the UVB-responsive genes (*HY5* and *CHS*) and accumulating UV-absorptive compounds. In addition, we also discovered that *SlUVR8* promotes fruit chloroplast development through enhancing accumulation of transcription factor GOLDEN2-LIKE2 (SlGLK2) which determines chloroplast and chlorophyll levels. Furthermore, UV-B radiation could increase expression of *SlGLK2* and its target genes in fruits and leaves. *SlUVR8* is required for UVB-induced *SlGLK2* expression. Together, our work not only identified the conserved functions of *SlUVR8* gene in response to UV-B stress, but also uncovered a novel role that *SlUVR8* could boost chloroplast development by accumulating SlGLK2 proteins.

## Introduction

Sunlight provides the energy of photosynthesis in sessile plants and also plays an essential role in regulation of their entire life cycle. However, ultraviolet-B (UV-B) light, as an indispensible component of sunlight, can retard plant growth by causing DNA damage, generating reactive oxygen species, and inhibiting photosynthesis^1^. To survive in sunlight, plants have to evolve the specific mechanisms perceiving and responding to the UV-B radiation^2,3^.

Recent studies revealed that UV RESISTANCE LOCUS8 (UVR8) protein was responsible for UV-B perception and signal transduction in *Arabidopsis*^1,4–6^. The UVR8 gene was first identified in a mutation screen for UV-sensitive plants and the *uvr8* mutant was hypersensitive to UV-B radiation^7^. The abolished UV acclimation of *uvr8* mutant is caused by failure of UV-induced expression of defense genes involved in UV damage repairment and UV protection, such as chalcone synthase (CHS) gene which is the committing enzyme for UV-absorptive flavonoid and anthocyanin biosynthesis^7,8^. Further investigations revealed that the transcription factor ELONGATED HYPOCOTYL5 (HY5) was a fundamental factor of UV signal pathway^9–11^ and UVR8 regulated *HY5* expression through physical association with chromatin in its promoter region^8^. Besides, UVR8-mediated signal facilitates HY5 and its homolog HYH binding to a T/G-box cis-acting element in the promoters of the UV-responsive genes^12^.

UVR8 forms homodimers in cytoplasm and their instant monomerization, which requires two tryptophan residues serving as the UV-B chromophore, can be activated by UV-B radiation^2,4,6,13^. The monomerized UVR8 proteins are translocated from cytoplasm to the nucleus for fulfilling its function and signal transduction^14,15^. UVR8 protein interacts with multifunctional E3 ligase CONSTITUTIVELY PHOTOMORPHOGENIC 1 (COP1) protein, a key regulator of light signaling, which is also involved in response to UV-B^14,16–19^. In the dark, COP1 interacts with DAMAGED DNA BINDING PROTEIN 1 (DDB1) and CULLIN4 (CUL4) to form the super complex of CUL4-DDB1-COP1-SPA E3 ligase which inhibits the photomorphogenesis by targeting HY5 for degradation. Under UV-B radiation, COP1-SPA complex disassociates from CUL4-DDB1 and interacts with monomerized UVR8^20^ to form UVR8-COP1-SPA complex which plays a positive role in stabilizing HY5 protein and its activity^21^. It appears that UVR8 modulates plant response to UV-B through regulating key transcription factor HY5 at both transcriptional and posttranslational level.

DDB1 was first demonstrated to be involved in damaged DNA repair, since it binds to the UV-induced DNA lesions and mediates nucleotide excision repair processes^22^. In Arabidopsis, DDB1 is associated with CUL4 and additional substrate receptor proteins including COP1 to form CUL4-RING ubiquitin ligase (CRL4) which is required for many cellular processes^23–25^. In tomato (*Solanum lycopersicum*), CUL4-DDB1 complex was proved to participate in plastid development and secondary metabolism^26–28^, epigenetic regulation^29^, and stress response^30–32^. The mutant *high pigment -1* (*hp1*), which is caused by a point mutation in tomato *DDB1* (*SlDDB1*) gene, displays the enhanced fruit nutrient contents resulting from the increased plastid (chloroplast) numbers and sizes in fruit cells^26^. Genetic suppression of tomato *CUL4* (*SlCUL4*) and *SlDDB1* genes resulted in the phenocopy of the *hp1* mutant^27^. These studies suggested that CUL-DDB1 complex plays a crucial role in plastid development in tomato. A transcription factor GOLD2-LIKE (SlGLK2), which determines plastid and chlorophyll levels by enhancing photosynthesis gene expression and chloroplast development^33–35^, is a target of CRL4 ubiquitin E3 ligase^36^. The degradation of SlGLK2 protein is impaired in the *hp1* mutant and *SlCUL4* silencing plants^28^.

Although the homologous genes of Arabidopsis *UVR8* were cloned in several plant species including Arabidopsis, *Populus euphratica*^37^, apple^38^, grape berry^39^, grapevine^40^, radish sprouts^41^, and *Chlamydomonas reinhardtii*^42^, little is known about characterization of tomato *UVR8* (*SlUVR8*) gene. In this study, we cloned *SlUVR8* gene and confirmed its conserved role in response to UV-B radiation. In addition, our results also revealed that SlUVR8 could mediate fruit plastid development under UV-B radiation, possibly through regulating the accumulation of transcription factor SlGLK2.

## Results

### Cloning of tomato UVR8 gene

We BLAST in tomato (*Solanum lycopersicum*) genome sequence database (https://sgn.cornell.edu/organism/Solanum_lycopersicum/genome) with Arabidopsis UVR8 protein sequence and got only one positive hit (Solyc05g018620), termed as *SlUVR8*, which suggested that the tomato genome contained only one homologous gene of *UVR8*. We cloned the gene by RT-PCR and the encoding protein shared 79% identities with Arabidopsis UVR8 (Supplemental Fig. 1). SlUVR8 also contains multiple repeated RCC1 domains, similar to UVR8 protein in Arabidopsis. The phylogenetic analysis indicated that SlUVR8 shared the best amino acid identity with *Solanum tuberosum* UVR8 (StUVR8) (Supplemental Fig. 2).

**Figure 1:**
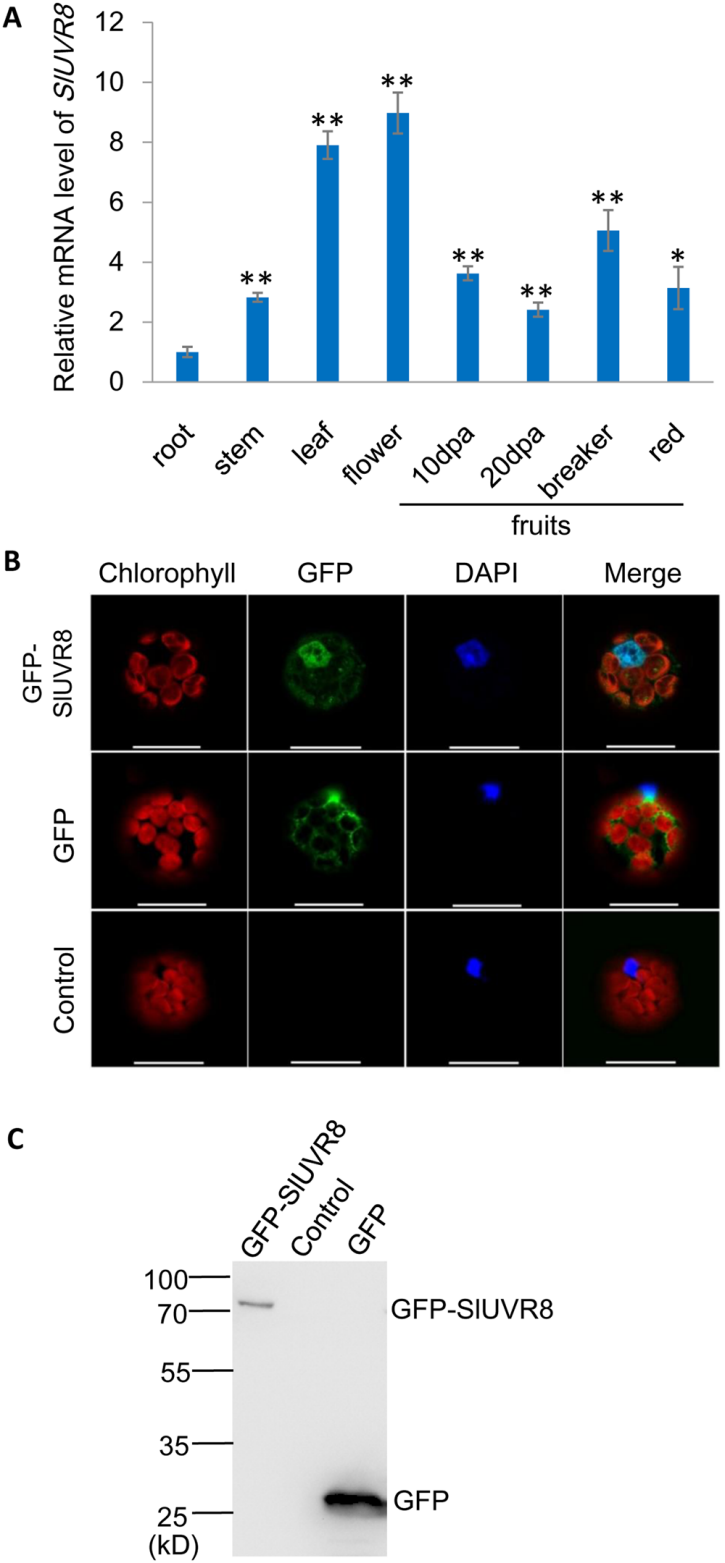
The expression pattern and sub-cellular localization of SlUVR8. (**A**) Constitutive expression of tomato *UVR8* in various tissues. The mRNA levels for tomato *UVR8* was analyzed by quantitative RT-PCR. Total RNAs were extracted from roots, stems, leaves, flowers and fruit pericarps at various developmental stages (10, and 20 days post-anthesis, breaker and ripe, respectively). The roots were harvested from plant grown indoor (22–28 °C, 16 h light and 8 h dark) and the rest tissues were harvested from plant grown in the outdoor field. Each bar represents mean value from three biological replicates from each type of tissues (n = 3). Error bars representing standard deviations (SD) are shown in each case. “*” and “**” means P < 0.05 and P < 0.001 respectively (Student’s *t* test). (**B**) Localization of GFP-UVR8 fusion protein transiently expressed in tobacco protoplasts. Upper panels, GFP-UVR8; middle panels, GFP; bottom panels, an untransformed protoplast as a negative control. Left to right: red, chlorophyll autofluorescence; green, GFP fluorescence; blue, nucleus stained with DAPI; merged, combined fluorescence from GFP, chlorophyll and DAPI. Scale bars = 25 μm. (**C**) Western-blot analysis of transient expression samples from (**B**) by using anti-GFP antibody. The positions of protein ladders were marked on the left side of the gel figure.

The *SlUVR8* gene was expressed constitutively in all the organs of tomato plants we tested. The expression levels in the leaves and flowers were apparently higher than that in other tissues, as indicated by quantitative RT-PCR (qRT-PCR) analysis (Fig. 1A). To check the sub-cellular localization of SlUVR8 protein, the gene of Green Fluorescent Protein (GFP) was fused with *SlUVR8* gene and GFP-SlUVR8 construct was transformed in protoplasts of tobacco leaves. The transformed protoplasts were observed by confocal microscope. As shown in Fig. 1B, GFP-SlUVR8 proteins were localized in the nucleus and cytoplasm simultaneously, similar to the sub-cellular localization of Arabidopsis UVR8^14,15^. Western-blot analysis indicated the both GFP and GFP-SlUVR8 were really expressed in the transformed protoplasts (Fig. 1C).

### SlUVR8 is required for tomato acclimation to UV-B radiation

**Figure 2:**
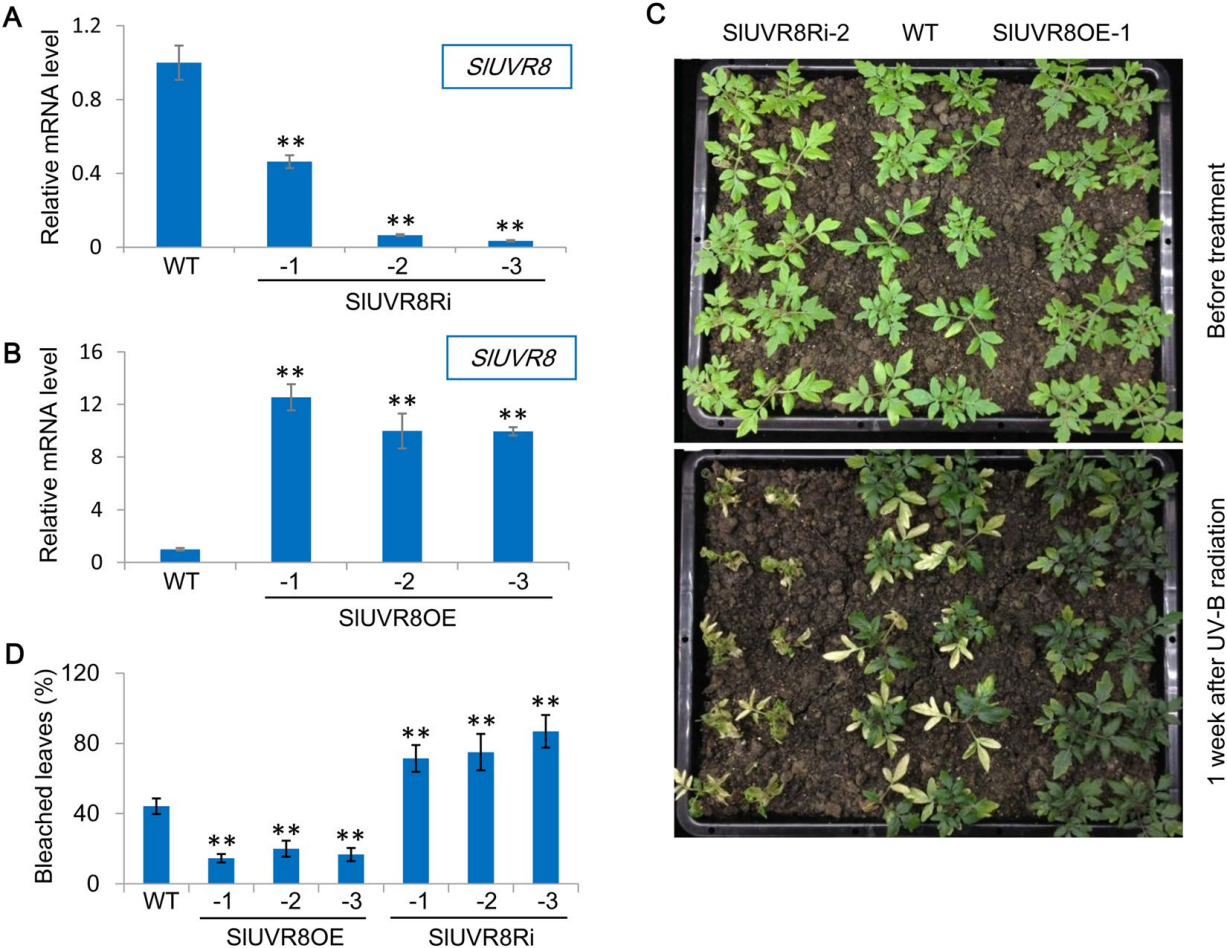
*SlUVR8* is required for tomato acclimation to UV radiation. (**A**) Quantitative RT-PCR analysis of *SlUVR8* mRNA levels in young leaves from wild-type Ailsa Craig (*WT*) and three independent *35S::SlUVR8Ri* transgenic lines. Each bar represents mean value from three biological replicates from each line (n = 3). Error bars representing SD are shown in each case. “*” and “**” means P < 0.05 and P < 0.001 respectively (Student’s *t* test). (**B**) Quantitative RT-PCR analysis of *SlUVR8* mRNA levels in young leaves from wild-type Ailsa Craig (*WT*) and three independent *35S::SlUVR8* transgenic lines. Each bar represents mean value from three biological replicates from each line (n = 3). Error bars representing SD are shown in each case. “*” and “**” means P < 0.05 and P < 0.001 respectively (Student’s *t* test). (**C**) Upper panels, phenotypes of 4-week-old *35S::SlUVR8Ri*-2, Ailsa Craig (*WT*), and *35S::SlUVR8OE*-1 seedlings grown under white light without UV-B. Bottom panels, phenotypes of the seedlings after UV-B radiation for a week. (**D**) Percentage of bleached leaves of the seedlings after UV-B radiation in (C). For each line, total 50 leaves from 10 different plants were counted (n = 50). “*” and “**” means P < 0.05 and P < 0.001 respectively (Student’s *t* test).

To study the function of *SlUVR8*-encoded protein and to explore its physiological role in UV-B response, two different kinds of transgenic tomato lines were generated by *Agrobacterium tumefaciens*-mediated transformation. One was the repression lines by using RNA interference (SlUVR8Ri), expressing *SlUVR8*-derived inverted-repeat sequences under the direction of the CaMV 35S promoter. The other was over-expression lines of *SlUVR8* (SlUVR8OE), also driven by 35S promoter. After screening the T0 generation plants with the quantitative RT-PCR (qRT-PCR) assays of *SlUVR8* expression, three independent lines were chosen for SlUVR8Ri (-1, -2, and -3) and SlUVR8OE (-1, -2, and -3), respectively. The homolozygous plants for each line were obtained in T2 generation. As validated by qRT-PCR, *SlUVR8* expression were significantly decreased in SlUVR8Ri lines (Fig. 2A) and remarkably increased in SlUVR8OE lines (Fig. 2B) compared to wild type (WT) plants.

The growth and development of transgenic plants were indistinguishable from WT plants in absence of UV-B light (Fig. 2C). After additional treatment with UV-B radiation for 1 week, however, SlUVR8Ri plants showed hypersensitive phenotypes including retarded growth, curly and bleached leaves, and even premature cell death (Fig. 2C) while SlUVR8OE plants displayed much less bleached leaves than WT plants (Fig. 2C and D).

**Figure 3:**
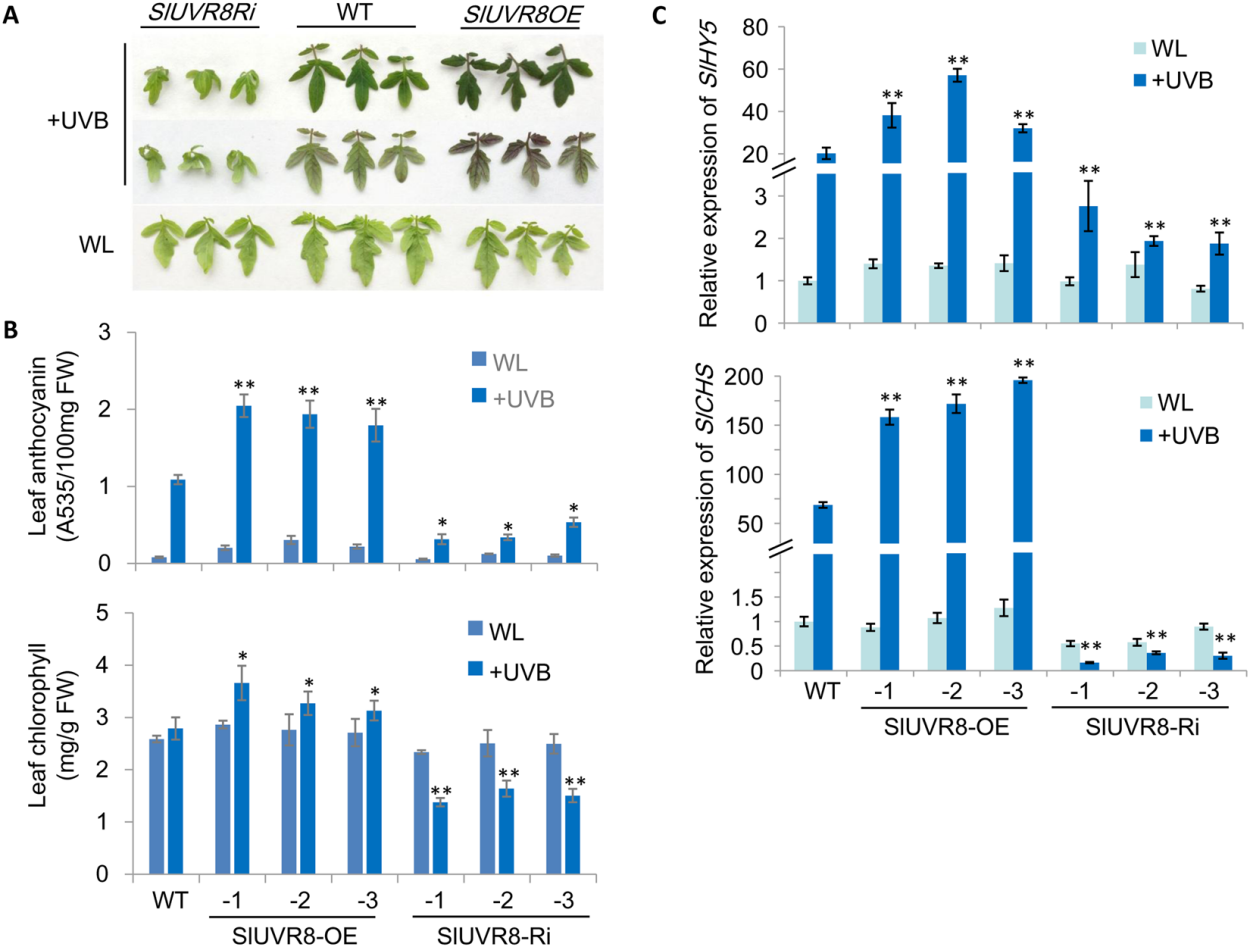
*SlUVR8* mediates UV-induced gene expression and anthocyanin accumulation. (**A**) Leaves detached from 30-d-old *35S::SlUVR8Ri*, Ailsa Craig (*WT*), and *35S::SlUVR8OE* seedlings grown under white light (photoperiod: 16 h light and 8 h dark) with or without 3-days UV-B radiation (12 h/day). (**B**) Quantitative measurement of anthocyanin and chlorophyll content in the fully expanded leaves in (A). Values were shown as “means ± SD”; Error bars represent SD of ten biological replicates. “*” and “**” means P < 0.05 and P < 0.001 respectively (Student’s *t* test). (**C**) The quantitative RT-PCR analysis of UVB-induced genes *SlHY5* and *SlCHS* of the seedlings in (A). Values were shown as “means ± SD”; Each bar represents mean value from three biological replicates from each line (n = 3). “**” means P < 0.001 (Student’s *t* test).

In addition, anthocyanin and chlorophyll accumulation were significiantly reduced in the leaves of SlUVR8Ri plants after UV-B treatment (Fig. 3A and B). However, SlUVR8OE plants enhanced accumulation of anthocyanin and chlorophyll in leaves (Fig. 3A and B). Moreover, qRT-PCR assays indicated that expression of tomato *HY5* (*SlHY5*), as well as *CHS* (*SlCHS*) gene, was down-regulated in SlUVR8Ri plants and up-regulated SlUVR8OE plants (Fig. 3C) compared with WT. Together, these results demonstrated that *SlUVR8* was required for tomato acclimation to UV-B radiation. Over-expression of SlUVR8 could increase plant tolerance to UV-B stress by up-regulating *SlHY5* expression and enhancing anthocyanin accumulation.

### SlUVR8 affects chloroplast development and nutrient quality of tomato fruits

**Figure 4:**
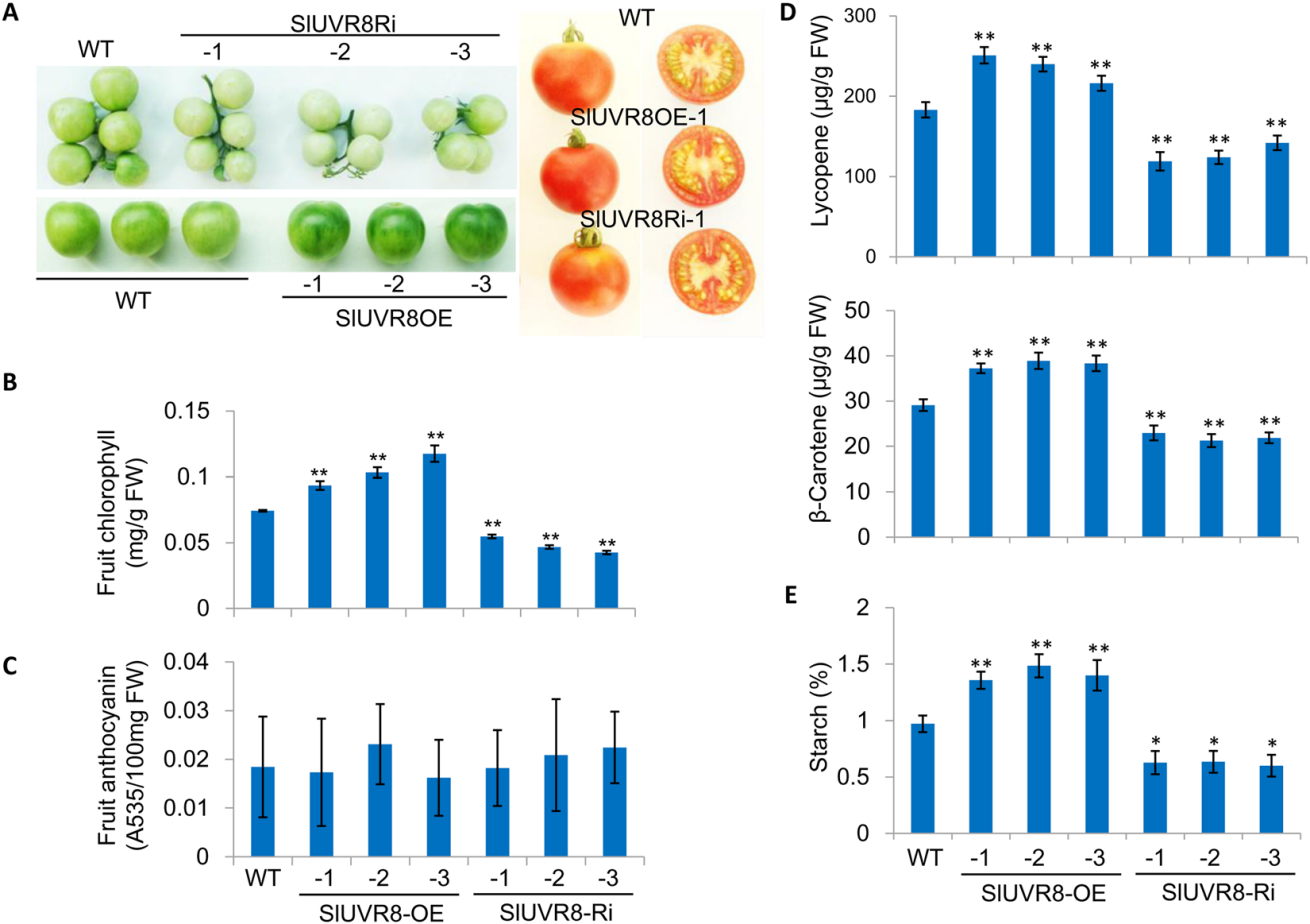
*SlUVR8* affects tomato fruit pigment and nutrient quality. (**A**) Phenotypes of immature fruits (25 DPA) and red ripe fruits from field-grown plants of wild-type Ailsa Craig (*WT*), *35S::SlUVR8Ri* lines and *35S::SlUVR8OE* lines. (**B**) Total chlorophyll levels from immature green fruit in (A). Values were shown as “means ± SD”; Error bars represent SD of 10 biological replicates. “**” means P < 0.001 (Student’s *t* test). (**C**) Lycopene, β-carotene, and starch levels in Ailsa Craig (*WT*), *35S::SlUVR8Ri* and *35S::SlUVR8OE* fruit. Lycopene and β-carotene levels were measured in red ripe fruits. Starch was measured in immature green fruits. Values were shown as “means ± SD”; Error bars represent SD of 10 biological replicates. “*” and “**” means P < 0.05 and P < 0.001 respectively (Student’s *t* test).

We grew the transgenic lines and WT plants in the outdoor fields, as described in “Materials and Methods”, with exposure to the natural sunlight. The SlUVR8Ri plants displayed normal growth without any phenotypes of hypersensitivity to UV radiation (data not shown). However, the SlUVR8Ri plants had pale green immature fruits (Fig. 4A). In contrast, SlUVR8OE plants had darker green fruits compared to WT plants (Fig. 4A). Accordingly, ripe fruits expressing *SlUVR8* had a 20% increase in chlorophyll content (Fig. 4B) and a 25% increase in carotenoids (Lycopene and β-Carotene) contents (Fig. 4D), as compared with WT fruits. In addition, starch accumulation were boosted in fruits of SlUVR8OE plants but reduced in SlUVR8Ri plants (Fig. 4E). However, the anthocyanin contents in fruits were very low and indistinguishable between WT and transgenic fruits (Fig. 4C). The previous studies revealed that low accumulation of flavonoids (including anthocyanins) in tomato fruits was caused by low expression of *chalcone isomerase* (*CHI*) gene^43^ and other genes^44^ required for anthocyanin biosynthesis. Our results indicated that overexpression of *SlUVR8* had no apparent effect on anthocyanin accumulation in tomato fruits, although *SlHY5* expression was also up-regulated in fruits of SlUVR8OE plants (Supplemental Fig. 3).

**Figure 5:**
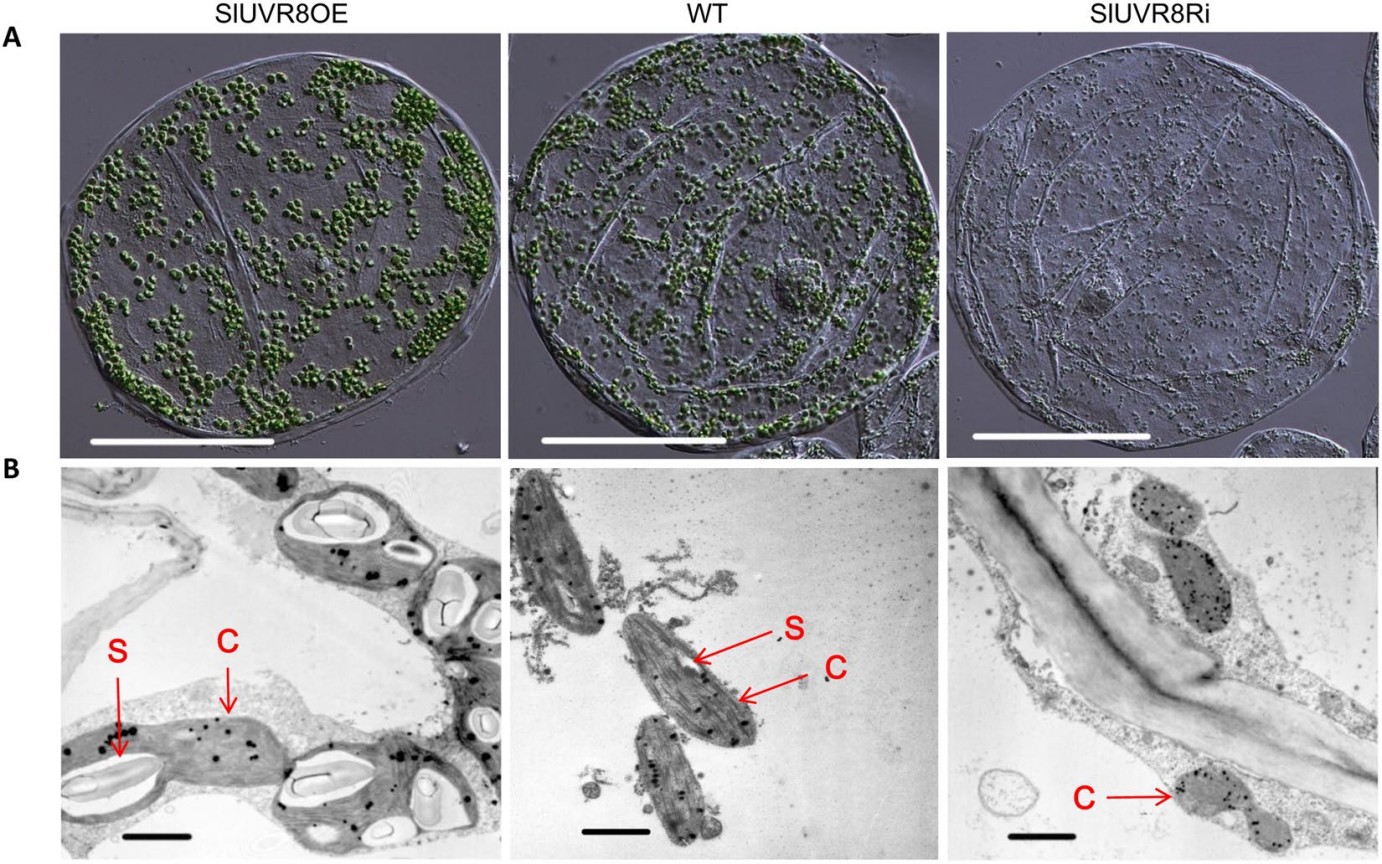
*SlUVR8* affects chloroplast development in fruit cells. (**A**) Isolated pericarp cells from immature green tomato fruits (25 DPA) of *SlUVR8OE*, wild-type (*WT*) and *SlUVR8Ri* plants grown in the outdoor field. Bars = 50 μm. (**B**) Transmission electron microscopy images of immature green fruit (25 DPA) chloroplasts from *SlUVR8OE*, wild-type (*WT*) and *SlUVR8Ri* plants grown in the outdoor field. Bars = 1 μm. Red “C” indicated Chloroplasts, Red “S” indicated Starch in chloroplast.

Meanwhile, we investigated chloroplast compartment sizes in pericarp cells of mature green fruits from WT and transgenic plants. The results indicated that plastid number per cell showed no substantial difference between WT and transgenic plants (Table 1), but plastid size (plastid plan area) was altered significantly (Table 1 and Fig. 5A). Larger chloroplasts were developed in fruit pericarp cells of SlUVR8OE plants while smaller chloroplast were displayed in SlUVR8Ri plants, as observed by optical microscope (Fig. 5A) and transmission electron microscope (Fig. 5B). The quantitative measurements confirmed our observations (Table 1). These results revealed that SlUVR8 could influence chloroplast size but not chloroplast number in tomato fruits. In addition, bigger starch grains were observed in chloroplasts from SlUVR8OE fruits while SlUVR8Ri fruits showed on obvious starch grains, as indicated by note “S” in Fig. 5B, in chloroplasts. This observation was consistent with our previous quantitative measurements of total starch content (Fig. 4D). It was noteworthy that we observed much less thylakoid stacks (grana) in the chloroplasts of SlUVR8Ri plants than in WT and SlUVR8OE plants (Fig. 5B), which implied that thylakoid membrane or photosynthesis apparatus in SlUVR8Ri plants might be damaged by solar UV radiation.

**Table 1:** Cell and plastid characteristics of fruit pericarp cells from tomato cv. Ailsa Craig (WT), SlUVR8OE and SlUVR8Ri transgenic plants. Values are means ± SE. Cell index is calculated as total plastid area per cell plan area. Immature green fruit were studied at 25d post-anthesis.

	Cell plan area (μm^2^, n = 30)	Plastid number per cell (n = 30)	Total plastid area per cell	Plastid density (per μm^2^ cell plan area, n = 30)	Plastid plan area (μm^2^, n > 1000)	Cell index
*WT*	57579 ± 3707	817 ± 58	10348 ± 811	0.014	12.7 ± 0.8	0.180
*UVR8-OE1*	56494 ± 5344	859 ± 65	13590 ± 799	0.015	15.8 ± 1.1	0.241
*UVR8-OE2*	58665 ± 4740	928 ± 67	13668 ± 785	0.016	14.8 ± 1.3	0.234
*UVR8-OE3*	55966 ± 4265	846 ± 50	12390 ± 829	0.015	14.7 ± 0.8	0.222
*UVR8-Ri1*	54370 ± 4505	942 ± 75	8368 ± 690	0.017	8.9 ± 0.6	0.154
*UVR8-Ri2*	56956 ± 5406	885 ± 83	7459 ± 789	0.016	8.4 ± 0.9	0.131
*UVR8-Ri3*	59592 ± 4893	872 ± 68	8782 ± 671	0.015	10.1 ± 0.9	0.148

### SlUVR8 enhances the accumulation of transcriptional factor SlGLK2 under UV-B radiation

**Figure 6:**
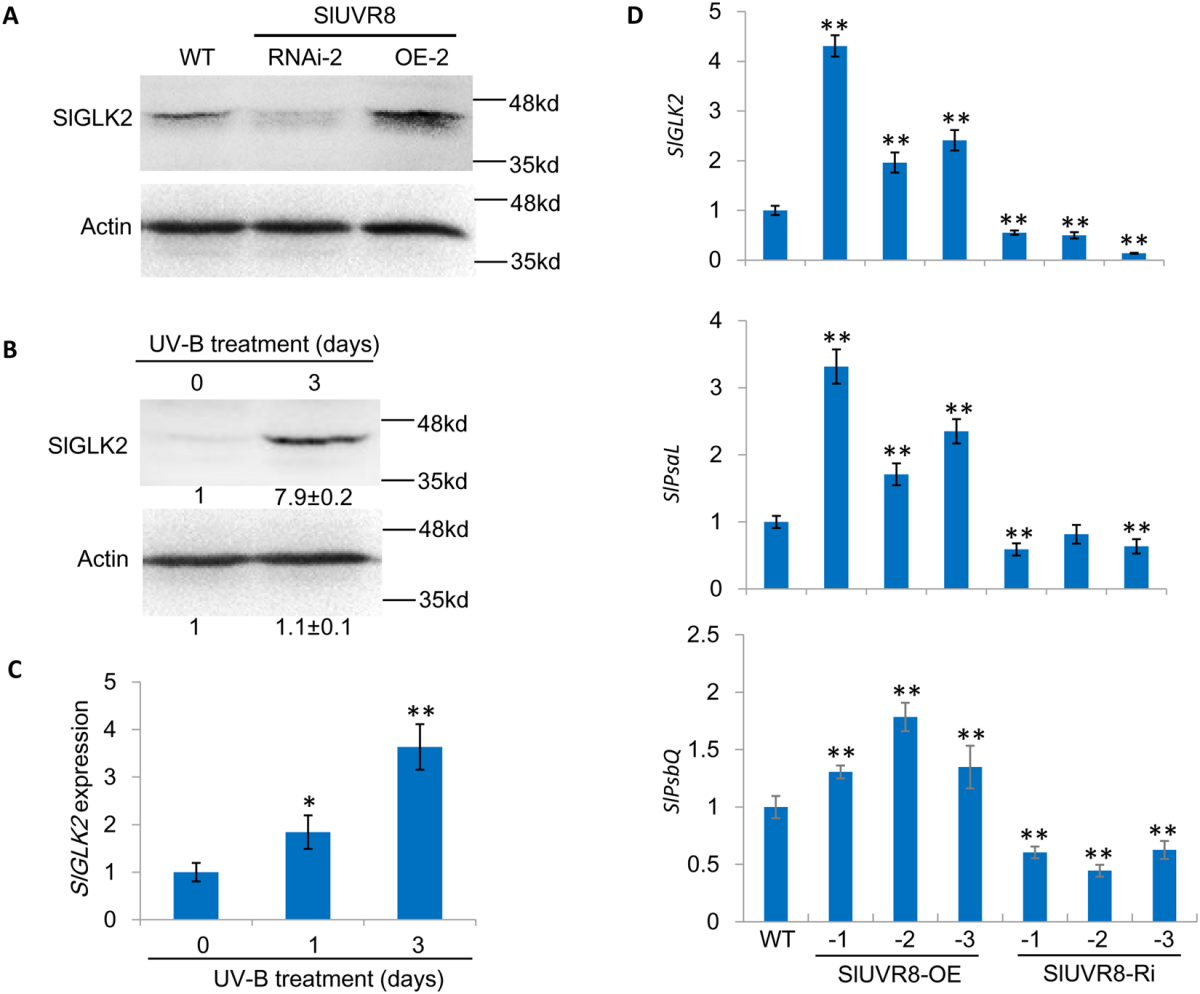
*SlUVR8* enhances SlGLK2 accumulation under UV radiation. (**A**) Immunoblot analysis of SlGLK2 levels among Ailsa Craig (*WT*), *35S::SlUVR8Ri* and *35S::SlUVR8OE* transgenic lines. The proteins extracted from immature fruits (25 DPA) from field-grown plants was resolved by SDS-PAGE, then probed with anti-SlGLK2 antibody and anti-β-actin antibodies. (**B**) Immunoblot analysis of SlGLK2 levels between Micro Tom (*WT*) plant grown in white light and plant grown in white light with added 3 days of UV-B irradiation. The proteins extracted from immature fruits (15 DPA) was resolved by SDS-PAGE, then probed with anti-SlGLK2 antibody and anti-β-actin antibodies. Blots were quantitatively analyzed by software ImageJ 1.46r. Values were shown as “means ± SD” from results of three independent experiments. (**C**) Quantitative RT-PCR analysis of *SlGLK2* mRNA levels in immature fruits (15 DPA) from Micro Tom (*WT*) with different UV-B irradiation time (0 d, 1 d and 3 d respectively). Values were shown as “means ± SD”; Error bars represent SD of 3 biological replicates. “*” and “**” means P < 0.05 and P < 0.001 respectively (Student’s *t* test). (**D**) Quantitative RT-PCR analysis of chloroplast development related genes *SlGLK2*, *SlPsa*L and *SlPsbQ* in Ailsa Craig (*WT*), 35S::SlUVR8Ri and 35S::SlUVR8OE transgenic lines. Total RNAs were extracted from fruit pericarps of immature fruits (25 DPA) from field-grown plants. Values were shown as “means ± SD”; Error bars represent SD of 3 biological replicates. “*” and “**” means P < 0.05 and P < 0.001 respectively (Student’s *t* test).

Tomato *GOLDEN2-LIKE* (*GLK*) transcription factor *SlGLK2* determines chlorophyll accumulation and chloroplast development in fruits through enhancing photosynthesis gene expression^28,33,34^. Therefore, we checked the SlGLK2 protein abundance in the fruits of transgenic plants grown under sunlight in open fields by using a SlGLK2-specific antibody which doesn’t recognize SlGLK1^28^. As shown in Fig. 6A, SlGLK2 proteins were over-accumulated in SlUVR8OE plants but less accumulated in SlUVR8Ri plants grown under sunlight. In addition, UV-B radiation could enhance the accumulation of SlGLK2 in fruits of WT plants grown in chamber with white light (Fig. 6B). These results indicated that *SlUVR8* might affect chloroplast development through targeting transcription factor SlGLK2. Next, the qRT-PCR assays indicated that UV-B could increase the mRNA levels of *SlGLK2* gene in WT fruits (Fig. 6C). It was noteworthy that UV-B radiation could increase SlGLK2 protein abundance by almost 7 times while mRNA level of SlGLK2 gene was increased by only 3 times (Fig. 6B and C). These results suggested that there possibly was a post-translational regulation in UV-B enhanced SlGLK2 accumulation. Also, *SlGLK2* expression was increased in fruits of SlUVR8OE plants but decreased in fruits of SlUVR8Ri plants (Fig. 6D). We also randomly selected two SlGLK2-targeted genes (*SlPsaL* and *SlPsbQ*), which encode photosystem I subunit L and photosystem II subunit Q respectively, and checked their expression in fruits of transgenic plants (Fig. 6D). The results indicated that over-expression of *SlUVR8* could increase the expression of transcriptional factor *SlGLK2* and its target genes, while silencing of *SlUVR8* led to suppression of *SlGLK2* and its target genes in fruits, when plants were exposed to the sunlight.

**Figure 7:**
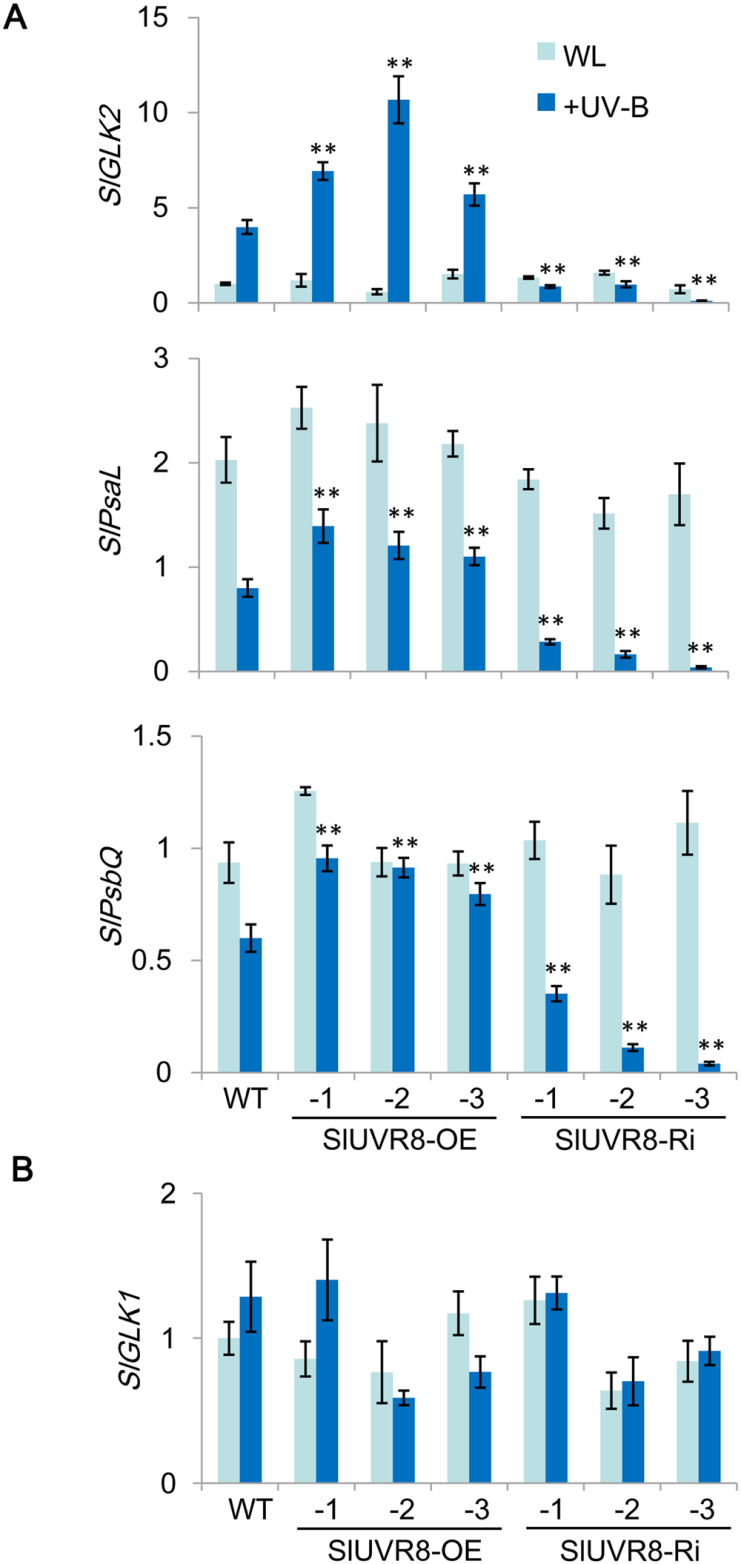
*SlUVR8* is required for UV-induced *SlGLK2* expression. (**A**) Quantitative RT-PCR analysis of chloroplast development related genes *SlGLK2* and its target genes (*SlPsa*L and *SlPsbQ*) in Ailsa Craig (*WT*), 35S::SlUVR8OE and 35S::SlUVR8Ri transgenic lines with or without 3-d UV-B irradiation. Total RNAs were extracted from totally expanded leaves of 30-d old seedlings grown in white light. Values were shown as “means ± SD”; Error bars represent SD of 3 biological replicates. “*” and “**” means P < 0.05 and P < 0.001 respectively (Student’s *t* test). (**B**) Quantitative RT-PCR analysis of *SlGLK1* gene in the same samples as described in (A). “**” means P < 0.001 (Student’s *t* test).

Since *SlGLK2* was also expressed in leaves^33,34^, we also checked its expression in leaves of transgenic plants with or without UV-B treatment. The qRT-PCR assays indicated that, when plants were cultured in indoor chamber with white light, *SlUVR8* had no apparent effect on expression of *SlGLK2* and its target genes (Fig. 7A). However, after UV-B treatment, *SlGLK2* expression was significantly increased in WT and SlUVR8OE leaves but not in SlUVR8Ri leaves. In addition, the expression of the target gene *SlPsaL* and *SlPsbQ* showed no obvious difference under white light but was suppressed severely after UV-B treatment in the leaves of SlUVR8Ri plants compared with WT and SlUVR8OE plants (Fig. 7A). These results confirmed that *SlUVR8* was required for UVB-enhanced expression of *SlGLK2* and its target genes. We also checked the expression of *SlGLK1* gene which was expressed only in leaves but not in fruits^33,34^. The qRT-PCR analysis indicated that UV-B radiation, as well as *SlUVR8* expression, had no significant effect *SlGLK1* expression in leaves (Fig. 7B).

## Discussion

As UV-B photoreceptor, *UVR8* gene has been characterized in several plant species including Arabidopsis, *Populus euphratica*^37^, apple^38^, grape berry^39^, grapevine^40^ and radish sprouts^41^, and in *Chlamydomonas reinhardtii*^42^. Here, we isolated tomato *SlUVR8* gene and identified its functions in plant’s adaptation to UV-B radiation. Transgenic evidences suggested overexpression of *SlUVR8* increased plant tolerance whereas silencing *SlUVR8* gene led to the hypersensitivity to UV-B stress (Fig. 2). In accordance with the previous studies in Arabidopsis, UV-B-induced *HY5* and *CHS* expression, as well as anthocyanin accumulation, were impaired in SlUVR8Ri plants but were enhanced in SlUVR8OE plants compared to WT (Fig. 3). These results demonstrated that SlUVR8 played an essential role of orchestrating expression of key UV-responsive genes (such as *SlHY5* and *SlCHS*) and accumulating UV-absorptive compounds with sunscreen functions of protecting plants from UV damage.

Sunlight provides energy for photosynthesis of chloroplast. However, solar UV radiation could damage photosynthesis and chloroplasts as well^45^. Numerous studies revealed that photosynthetic apparatus was susceptible to damage caused by UV-B^46–50^. The adverse effects from enhanced UV-B radiation on chloroplasts include but not limited to loss in integrity of the thylakoid membranes^51^, impaired activity of photosystem II (PSII)^52,53^, enhanced protein degradation^50^, inhibited carbon fixation^54^ and reduced content of starch and chlorophyll^53,55^. Therefore, plants must employ some strategies or mechanisms, some of which remain to be elucidated yet, to respond to these problems and to maintain photosynthesis and chloroplast functions. A few studies suggested that UVR8 is required for maintaining the photosynthesis efficiency under enhanced UV-B radiation in Arabidopsis^49,50^. The underlying mechanism, however, has not been revealed yet. In our work, we proved that *SlUVR8* promote chloroplast development in tomato fruits when plants were grown in outdoor fields and exposed to natural sunlight (Fig. 4). Silencing *SlUVR8* caused some developmental issues in fruits including reduced size of chloroplasts and decreased contents of chlorophyll and starch, while overexpresssion of *SlUVR8* led to larger chloroplasts and increased contents of chlorophyll and starch (Fig. 5). The previous studies revealed that low accumulation of flavonoids (including anthocyanins) in tomato fruits resulted from low expression of *chalcone isomerase* (*CHI*) gene^43^ and other genes^44^ required for anthocyanin biosynthesis. Our results also showed that the anthocyanin contents in fruits were very low and indistinguishable between WT and transgenic fruits (Fig. 4C). It is conceivable that deficiency of anthocyanins was not responsible for chloroplast abnormality in fruit cells from SlUVR8Ri plants. In our case, SlUVR8 might play another role in affecting chloroplast development of tomato fruits rather than its role in accumulating UV-absorbing compounds. Therefore, we speculated that SlUVR8 could enhance chloroplast development, facilitate the recycling of damaged chloroplasts, and thereby contribute to maintain photosynthesis under UV-B stress.

In tomato, silencing *SlHY5* also caused abnormalities in both organization and abundance of thylakoids^26^. However, no reports yet showed that overexpression of *SlHY5* could lead to larger chloroplasts and high pigment of fruits in tomato. Therefore, we surmise that other genes are also involved in the UVR8-mediated chloroplast development. Our results disclosed that UV-B could enhance accumulation of SlGLK2, which was a key regulator of fruit chloroplast development^33,34^, and the UVB-enhanced SlGLK2 accumulation was dependent on *SlUVR8* gene. Several studies demonstrated that *SlGLK2* influenced chloroplast development and chlorophyll level by increasing fruit photosynthesis and chloroplast gene expression^33,34^. The accumulation of carbohydrates and carotenoids were elevated in fruits of *SlGLK2* overexpression plants. Our results showed that more chlorophyll, starch, and carotenoids were accumulated in fruits of SlUVR8OE plants (Fig. 4), similar to the phenotypes of fruits expressing *SlGLK2*. In addition, our results indicated that SlGLK2 proteins were over-accumulated in SlUVR8OE fruits but less-accumulated in SlUVR8Ri fruits under sunlight (Fig. 6). Further studies revealed that expression of *SlGLK2* and its randomly-selected target genes were elevated in SlUVR8OE plants under UV-B radiation (Figs 6 and 7). Silencing *SlUVR8* abolish UVB-induced expression of *SlGLK2* and its target gene (Figs 6 and 7). These results suggested that *SlUVR8* gene is required for UVB-enhanced *SlGLK2* expression. Therefore, we proposed an unknown pathway that photoreceptor *UVR8* promote chloroplast development in tomato under UV-B stress by enhancing accumulation of SlGLK2. Of course, we can’t exclude the role of *SlHY5* in UV-mediated chloroplast development^26^. A study about the root greening in Arabidopsis^56^ showed that *GLK2* could induce the accumulation of HY5 and combination of *GLK2* and *HY5* mediated the coordinated expression of many key genes of chloroplast biogenesis. This study might shed some light on the mechanism that both *SlHY5* and *SlGLK2* are possibly required for UVB-mediated chloroplast development. The previous study showed that expression of *GLK2* was also responsive to photooxidative chloroplast damage and retrograde signals from plastid in Arabidopsis^57^, indicating that *GLK2* might play an additional role in adjusting photosynthetic capacity in changing environmental conditions. It will be interesting to check whether silencing *SlGLK2* alone could affect UVB-mediated chloroplast development and plant response to UV-B stress in further investigations.

In Arabidopsis, transcription factor *HY5* is regulated by *UVR8* at both transcriptional and post-translational levels^8,17,21^. Here, our immunoblot analysis showed that UV-B radiation could increase SlGLK2 protein abundance by about 7 times while mRNA level of *SlGLK2* gene was increased by only 3 times, as compared with untreated condition (Fig. 6B and C). These results suggested that a post-translational modification, which requires further studies, might exist in this case. In Arabidopsis, UVR8 were associated with chromatin in the region of the *HY5* promoter and regulate *HY5* transcription^8,58,59^. However, a recent study^60^ indicated that UVR8 didn’t bind to chromatin *in vivo* or *in vitro*, possibly because of lacking critical histone- and DNA-interaction residues. Therefore, the exact mechanism of *UVR8* regulating transcription remains controversial and elusive. In our study, there is no evidence yet that SlUVR8 interacts with the promoter of *SlGLK2* gene directly. Therefore, we can’t exclude the possibility that SlUVR8 might regulate *SlGLK2* gene expression through another transcription factor or proteins. Further investigations are needed to validate our speculations and figure out the mysteries.

Together, we revealed that *SlUVR8* played a conserved role in plant acclimate to UV-B stress. Besides, our results indicated that *SlUVR8* mediated the fruit chloroplast development by enhancing accumulation of transcription factor SlGLK2. Moreover, manipulation of *SlUVR8* level represents a new option to enhance both tolerance to UV-B stress and nutrient values of tomato fruits, especially when plants were grown in outdoor fields with intense sunlight.

## Materials and Methods

### Plant Materials and Growth Conditions

Tomato (*Solanum lycopersicum*) cv Ailsa Craig (LA2838A) were acquired from the Tomato Genetics Resource Center (UC Davis, USA). Tomato plants were grown under artificial conditions (22–28 °C, 16 h light and 8 h dark) for several weeks and then transplanted into the outdoor field (located at the suburbs of Chengdu, China) for 4 months (from May to August). During this time, the maximum value of UV-B irradiance at noon (1 p.m.) is 9.5 μmol/m^2^/s and the minimum value is 4.3 μmol/m^2^/s, as measured by TBQ-ZW-2 UV-B (280–320 nm) light meter (Shanghai Minyin Electrics co., Ltd, China). For photomorphogenic UV-B treatment, seedlings were grown under white light (photoperiod: 16 h light and 8 h dark) supplemented with Philips TL20W/01RS narrowband UV-B tubes (23 μmol/m^2^/s) for 12 hours per day.

### Generation of transgenic tomato

The *Agrobacterium* EHA105 strains containing vectors pBI121-*SlUVR8* or pBI121-*SlUVR8*-*RNAi* were used to transform tomato cv Ailsa Craig by using *Agrobacterium tumefaciens*-mediated transformation^61^. Primary transformants (T0) and their offspring were cultivated under the artificial conditions for 4 weeks and then transplanted into the outdoor field (located at the suburbs of Chengdu, China). The T-DNA insertions of transgenic plants were identified by PCR using *NPTII*-specific primers and the expression of *SlUVR8* gene was validated by real-time RT-PCR using *SlUVR8*-specific primers. Three independent transgenic lines were used for phenotypic and molecular analysis.

### Quantitative RT-PCR analysis

Total RNAs were isolated using Trizol reagent (Invitrogen). The genomic DNAs in total RNAs were erased and first-strand cDNAs were synthesized with oligo-dT(18T) primers by using cDNA Synthesis kit, according to the manufacturer’s protocol (TransGen, Beijing, China). Quantitative PCR was conducted on the ABI StepOnePlus PCR System by using the TransStart Green qPCR SuperMix (TransGen). Gene expression was normalized by the expression of *UBI3* gene. All the primers used are listed in Table S1.

### Subcellular localization analysis

The coding sequence of the *SlUVR8* gene was cloned into the expression vector pART27-mcs:GFP and generated the pART27- GFP-SlUVR8 construct. Plasmid vectors pART27-GFP-SlUVR8 and pART27-GFP were introduced into protoplasts of tobacco (*Nicotiana benthamiana*) according to the methods described previously^62,63^. After incubation at 25 °C for 12–16 h, protoplasts were observed under a Leica TCS SPII confocal microscope using 488 and 633 nm excitation wavelengths and three-channel measurement of emission: 435 nm (blue/DAPI), 522 nm (green/GFP) and 680 nm (red/chlorophyll).

### Protein extraction and immunoblot analysis

Tissue was ground in liquid N_2_ and was treated in boil water for 5 min with SDS-PAGE loading buffer to extract total proteins. Immunoblot analysis was performed as described previously^64^. The SlGLK2-specific antibodies recognized the SlGLK2 N-terminal unique sequence (synthetic peptide SSSLSYKNERENYD, 5–18), but not SlGLK1 protein, as described in the previous study^28^. The secondary antibody Goat-anti-rabbit IgG conjugated to horseradish peroxidase (HRP) were purchased from Hangzhou HuaAn Biotechnology Co., Ltd.

### Plastid Analysis and Transmission Electron Microscopy

The fruit tissues used for plastid analysis were harvested from plants grown in outside open fields and processed as previously reported^65^. In brief, the fruit pericarp tissues were immersed in 3.5% glutaraldehyde for 1 hour at least, then incubated in 0.1 M Na_2_-EDTA for 30 min at 60 °C. The sliced and smashed samples were imaged with a Leica DM2500 microscope, and cell plan area was measured by Image-Pro Plus. For transmission electron microscopy, outer pericarp tissues were prefixed in 3% glutaraldehyde in 0.1 M phosphate buffer at 4 °C. Tissues were post fixed in 1% osmium tetroxide, dehydrated in series acetone, infiltrated in Epox 812 for a longer, and embedded. The semithin sections were stained with methylene blue and ultrathin sections were cut with diamond knife, stained with uranyl acetate and lead citrate. Sections were examined with a Transmission Electron Microscope (TEM; HITACHI, H-600IV, Japan).

### Anthocyanin, chlorophyll and carotenoid assays

Anthocyanin was assayed according to the procedures described previously^66^. chlorophyll and green fruits’ total carotenoid was extracted into 80% acetone, and their content was calculated by using Lichtenthaler’s formulas, in which chlorophyll a equals 12.21*A*_663_-2.81*A*_645_, chlorophyll b equals 20.13*A*_645_-5.03*A*_663_, total carotenoids equals (1000 *A*_470_-3.27*C*a-104*C*b)/229. The red fruits’ carotenoid contents were measured by HPLC as previously described^27^, with analytical reagent lycopene and β-carotene as standards.

### Starch Analysis

Starch quantification was determined using a starch assay kit (STA20; Sigma-Aldrich) following the manufacturer’s protocol^34^.

## Electronic supplementary material

Suplemental Figures and Table
