# Supplementary material for: Tomato UV-B receptor SlUVR8 mediates plant acclimation to UV-B radiation and enhances fruit chloroplast development via regulating SlGLK2
Source: Sci Rep. 2018 Apr 17;8:6097. doi: 10.1038/s41598-018-24309-y (PMC5904186; doi:10.1038/s41598-018-24309-y)
Supplement: Supplementary file 1 — Suplemental Figures and Table [file 41598_2018_24309_MOESM1_ESM.pdf]

Supplemental Figures and Table

Title of manuscript: Tomato UV-B receptor SlUVR8 mediates plant acclimation to UV-B radiation and enhances fruit chloroplast development via regulating SlGLK2

Authors: Huirong Li, Yuxiang Li, Heng Deng, Xiaochun Sun, Anquan Wang, Xiaofeng Tang, Yongfeng Gao, Ning Zhang, Lihuan Wang, Shuzhang Yang, Yongsheng Liu, and Songhu Wang

Figure legends:

Supplemental Figure 1. The aligned conserved domains of SlUVR8 protein

(A) Structural domains of the SlUVR8 and AtUVR8 proteins. Analysis of protein sequences in the National Center for Biotechnology Information (NCBI) database was performed using the CD-search software.

(B) Amino acid sequence alignment of UVR8 proteins from *Solanum lycopersicum, Arabidopsis, Nicotiana tomentosiformis*, *Populus trichocarpa*, and *Oryza sativa*. The alignment was constructed using the DNAMAN version 5.2.2 software, and highlighting was by Boxshade (http://ch.embnet.org/software/BOX_form.html). Identical residues are indicated by black boxes. Red dots above the sequences indicate the 14 conserved tryptophan residues and the red frames indicate the triad tryptophans (positions 233, 285, 337 in the Arabidopsis sequence) that form a pyramid arrangement with W94 on the adjacent monomer. Black dots above the sequences indicate the two arginine residues (positions 286, 338 in the Arabidopsis sequence) that are key to maintaining the AtUVR8 dimer. Red lines under the sequences indicate the conserved “GWRHT” motifs and the green box indicates the conserved C27 region. Blue lines indicate the seven blades compared with AtUVR8. Grey lines under the AtUVR8 sequence indicate the missing residues in the AtUVR8 crystal structure.

Supplemental Figure 2. The phylogenetic analysis of SlUVR8 protein

Phylogenetic analysis of UVR8 proteins of plants. The phylogenetic tree was constructed using MEGA4 via the neighbor-joining method. Bootstrap values from 1000 replicates were calculated and are indicated at branch points on the neighbor-joining tree. The tree includes SlUVR8(XP_010321071.1) from *Solanum lycopersicum*, StUVR8(CAC84597.2) from *Solanum tuberosum*, BvUVR8(XP_010695065.1) from *Beta vulgaris*, BpUVR8(AHY02156.1) from *Betula platyphylla*, BdUVR8(XP_003579788.1) from *Brachypodium distachyon*, BrUVR8(XP_009112108.1) from *Brassica rapa*, CsCUVR8(XP_010484148.1) from *Camelina sativa* (L.) Crantz, CsUVR8(XP_006464912.1) from *Citrus sinensis*, CmUVR8(XP_008442146.1) from *Cucumis melo*, EgJUVR8(XP_010930682.1) from *Elaeis guineensis Jacq*, EgUVR8(XP_010025746.1) from *Eucalyptus grandis*, MdUVR8(XP_008394185.1) from *Malus domestica*, MaUVR8(XP_009404858.1) from *Musa acuminates*, NnUVR8(XP_010270276.1) from *Nelumbo nucifera*, PmUVR8(XP_008227523.1) from *Prunus mume*, PbUVR8(XP_009355901.1) from *Pyrus x bretschneideri*, RcUVR8(XP_002522929.1) from *Ricinus communis*, NtUVR8(XP_009626007.1) from *Nicotiana tomentosiformis*, SiUVR8(XP_011099677.1) from *Sesamum indicum*, SiBUBR8(XP_004975662.1) from *Setaria italica* (L.) Beauv. var. germanica (Mill.) Schrad, ThUVR8(XP_010547681.1) from *Tarenaya hassleriana*, PeUVR8(XP_011022871.1) from *Populus euphratica*.

Supplemental Figure 3 SlUVR8 and SlHY5 expression in fruits from transgenic plants.

Real-time quantitative PCR analysis of *SlUVR8*, and *SlHY5* in Ailsa Craig (*WT*), *35S::SlUVR8Ri* and *35S::SlUVR8OE* transgenic lines. Total RNAs were extracted from fruit pericarps of immature fruits

(25 DPA) from field-grown plants. Error bars represent SD of 3 biological replicates. “*” and “**” means $P<0.05$ and $P<0.001$ respectively (Student’s *t* test).

Supplemental Figure 4 The uncropped gel of Figure 1C.

Red frame indicates the cropped position.

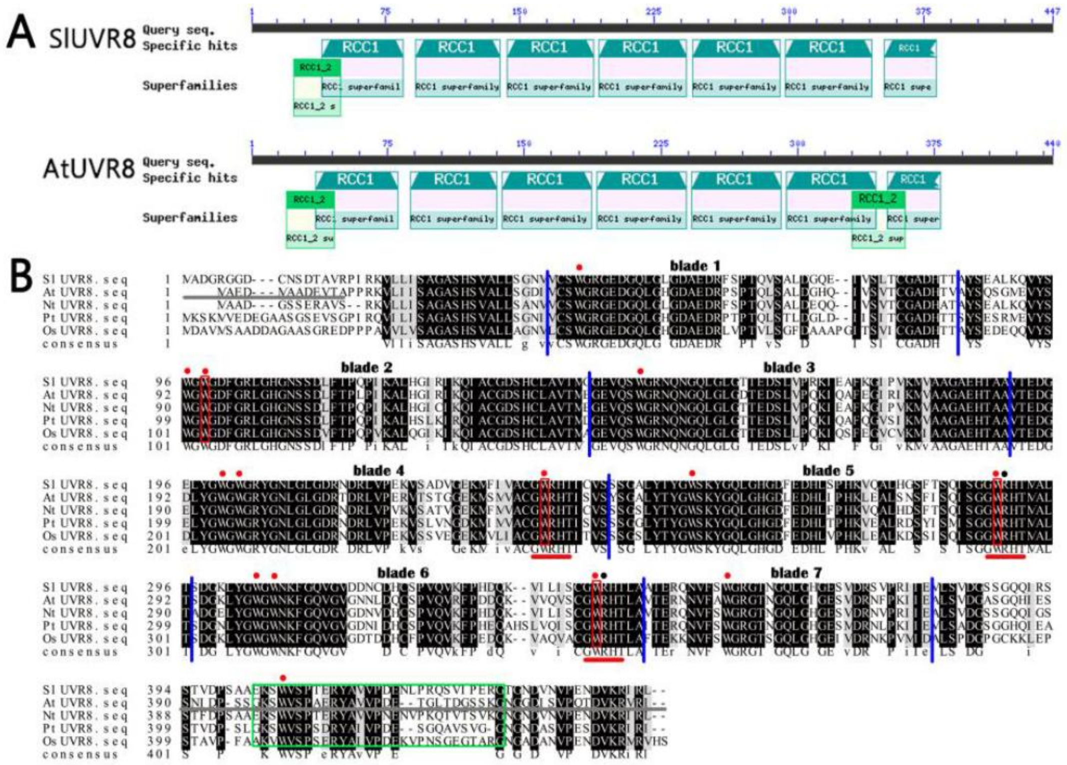

Supplemental Figure 1. The aligned conserved domains of SlUVR8 protein

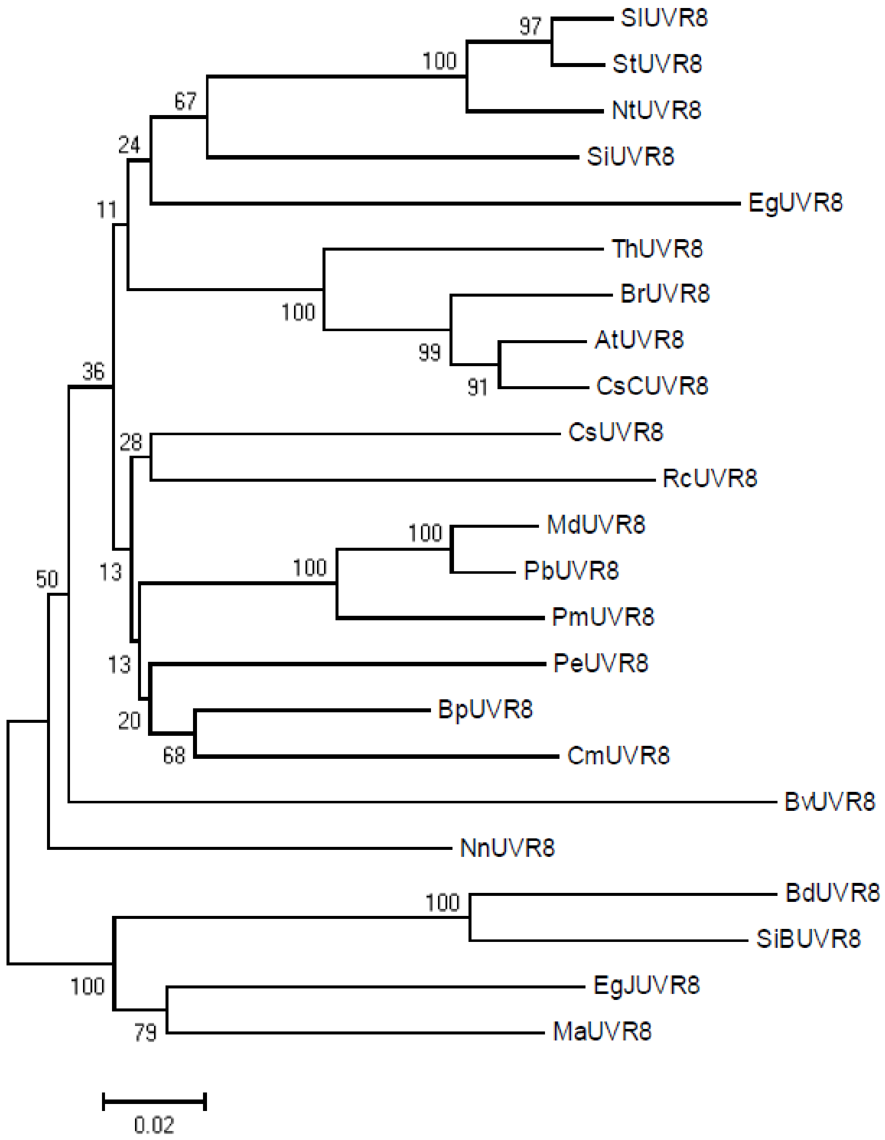

Supplemental Figure 2. The phylogenetic analysis of SlUVR8 protein

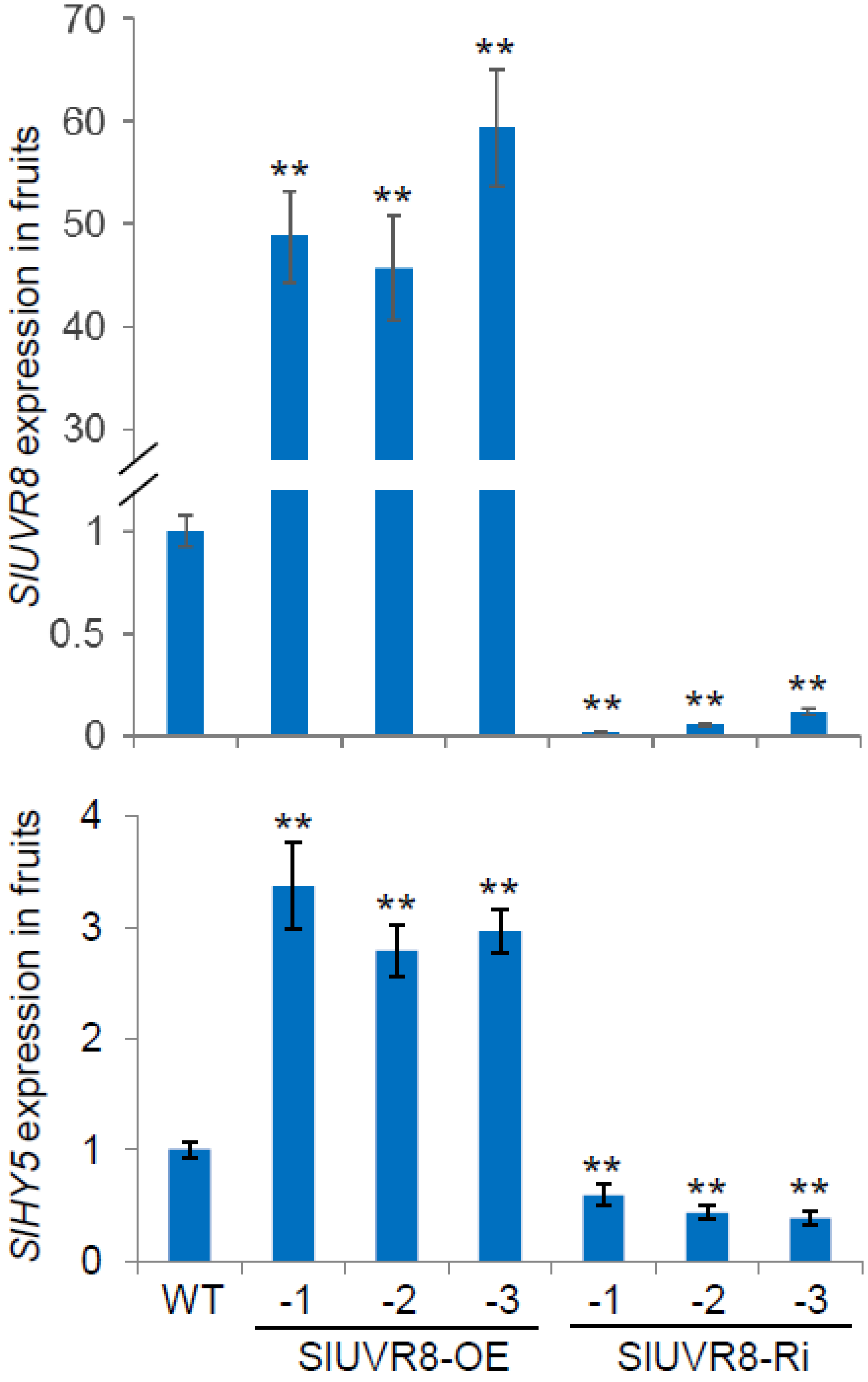

Supplemental Figure 3 SlUVR8 and SlHY5 expression in fruits from transgenic plants

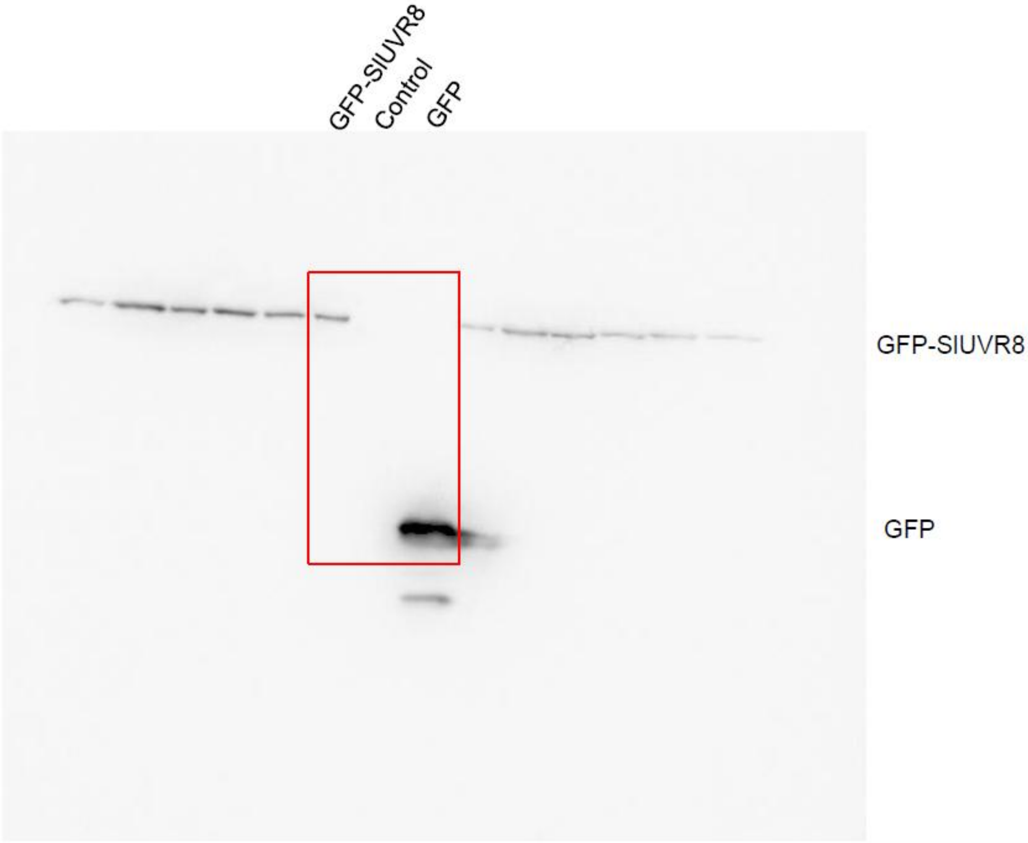

Supplemental Figure 4 The uncropped gel of Figure 1C

Primer	Sequence	Usage
SlUVR8outF	CCTTCCATAAAAAAAATTCC	gene-cloning
SlUVR8outR	AGTCCACCATCCACTGCTAG	gene-cloning
SlUVR8F1	TCTAGAATGGCGGACGGAAGAGGT	gene-cloning
SlUVR8R1	GAGCTCTCATAATCGGATCCTTTTCACA	gene-cloning
SlUVR8F2	GGTACCATGGCGGACGGAAGAGGT	GFP fusion protein
SlUVR8R2	CTCGAGTAATCGGATCCTTTTCACATCA	GFP fusion protein
SlUVR8rtimeF	GCAGAGTTGGGGGCGAAA	Real-time PCR
SlUVR8rtimeR	TTTCTCCCACATCGGCTGA	Real-time PCR
SlCHSrtimeF	TTTTGGTGATGGGGCGGC	Real-time PCR
SlCHSrtimeR	GCCTTCGCTATCGGGGACA	Real-time PCR
SlHY5rtimeF	AGCGACGAGTTCTATTGCCG	Real-time PCR
SlHY5rtimeR	GCTTCTCCGCCCATCTCC	Real-time PCR
SlCHLHrtimeF	TCTCAAACGCTTCGGGTTCTTA	Real-time PCR
SlCHLHrtimeR	TCAAAAACTTTCCTCTTCTCCATCA	Real-time PCR
SlPsbQrtimeF	TGGTCTTGTTGCTGCTGGC	Real-time PCR
SlPsbQrtimeR	CAAAGTTCCAGGCAATCCACC	Real-time PCR
SlPsaLrtimeF	CTTCACCGTCAAGGCTGTCC	Real-time PCR
SlPsaLrtimeR	GATAGGCAGGCAAGTTGGAGAG	Real-time PCR
SlGLK2rtimeF	CCTTACATGTTTGGGGGCATCCAC	Real-time PCR
SlGLK2rtimeR	GGGGTGCAAATCAGAGGC	Real-time PCR

Table S1 The primer used in this study
